# The Dust Separation Efficiency of Filter Bags Used in the Wood-Based Panels Furniture Factory

**DOI:** 10.3390/ma15093232

**Published:** 2022-04-29

**Authors:** Czesław Dembiński, Zbigniew Potok, Martin Kučerka, Richard Kminiak, Alena Očkajová, Tomasz Rogoziński

**Affiliations:** 1 Department of Furniture Design, Faculty of Forestry and Wood Technology, Poznań University of Life Sciences, 60-627 Poznań, Poland; czeslaw415@wp.pl (C.D.); zbigniew.potok@up.poznan.pl (Z.P.); 2 Department of Technology, Faculty of Natural Sciences, Matej Bel University in Banská Bystrica, Tajovského 40, 974 01 Banská Bystrica, Slovakia; martin.kucerka@umb.sk (M.K.); alena.ockajova@umb.sk (A.O.); 3 Department of Woodworking, Faculty of Wood Sciences and Technology, Technical University in Zvolen, 960 01 Zvolen, Slovakia; richard.kminiak@tuzvo.sk

**Keywords:** separation efficiency, wood dust, long-term filtration, dust filtration, maturation of filter bags

## Abstract

The relationship between the conditions of the use of filter bags made of non-woven fabric and the separation efficiency of wood dust generated in a furniture factory was experimentally determined in the conditions of pulse-jet filtration using a pilot-scale baghouse as waste during the processing of wood composites. The experiments were carried out, and we describe the results of the experiment as consisting in assembling one type of filter bag in two dust extraction installations operating under different operating conditions in the same furniture factory. The filter bags working in the assumed time intervals were then tested for their separation efficiency using a stand for testing filtration processes on a pilot scale. The test results are presented in the form of graphs and tables describing both the characteristics of the dust extraction installations and the filter fabric used, as well as the separation efficiency of bags used at different times in different industrial operating conditions for each of them. The conducted research allowed us to recognize the phenomenon of filtration in relation to a very important value, which is the separating efficiency of dust extraction in various operating conditions of dust extraction installations in a furniture factory during the long-term use of filter fabrics. The obtained results allowed us to determine the separation efficiency for the tested bags at a level of over 99.99% and to state that this separation efficiency increased with the working time of the bag. The structure of the outlet dust from filters in the wood composites processing factory constitutes an element of the working environment if the purified air is returned in a recirculation circuit to the interior of the working area. Thanks to this, it is possible to predict the separation efficiency in the long-term use of filter dust collectors for wood dust in furniture factories.

## 1. Introduction

Technological progress that is taking place in the furniture industry, in addition to comprehensive benefits, also brings about threats in the form of increasing interference with the natural environment. One of its expressions is the increasing demand for wood and its products [1,2,3,4]. The growing amount of wood consumption and wood products produced worldwide is directly related to the increase in dust emissions from wood processing and wood composites. These pollutants pose a very serious threat to human health. Wood dust is one of the most dangerous pathogens found inside factories processing wood materials [5,6,7,8,9,10]. The dimensions of the dust particles and their properties are conducive to long-term floating in the air, which is a very serious exposure for people staying in it as the exposure is a harmful factor to the human body. Therefore, the dust concentration in the air is an amount that should be systematically controlled, and it should be ensured that its level does not exceed the permissible concentrations [11,12,13,14,15]. In order to prevent the harmful effects of wood dust, the permissible amount in the air surrounding the woodworking stations has been determined. Until 2018, the permissible concentration of beech and oak wood dust in the inhaled air was 2 mg·m^−3^, and for the dust of other species, 4 mg·m^−3^. In 2018, the regulations were harmonized, and as a result of the compromise, the standard of wood dust concentration in the inhaled air for all wood species was 3 mg·m^−3^ [16]. From January 2022, the maximum permissible concentration of the inhalable fraction will be only 2 mg·m^−3^. To reduce the amount of wood dust in the air, it is worth paying attention to the sources of its formation and the phenomena occurring during air purification from dust particles. The amount of dust in the working environment under air recirculation conditions depends on an efficiently conducted filtration process. The quantity describing the filtration process is, apart from the filtration resistance, also the dust separation efficiency. In these works, various aspects related to the pulse-jet filter were investigated. Unfortunately, most of the work is not concerned with wood dust, which makes it necessary to conduct research to explain the phenomena occurring during the filtration of wood dust. Since it is a very complex process, it must be carefully directed. Problems related to dust filtration have been the subject of previous papers [17,18,19,20,21]. Increasing the filtration separation efficiency or the influence of the filter fabrics used has already been the subject of research [22,23]. However, it is difficult to find studies whose results would show the variability of filtration efficiency depending on the length of time the filter bags are used. In order to assess the filtration process and the air quality at the outlet of the filter, it is, therefore, necessary to know the ability of the filters to clean it with the assumed filtration efficiency in the period of long-term use in industrial conditions. This issue was investigated in the past by Thorpe and Brown [24], who undertook to investigate the emission and filtration efficiency of dust from hand sanders used in the wood industry, but their research was based on a process supervised in laboratory conditions, assuming that during the operation of the tested sander, attempts were made to simulate the conditions of industrial use. The researchers only had new filter materials at their disposal. However, the efficiency of filtration depends not only on the design of the filters and the type of filter fabric used in it but also on the length and conditions of use of the filters in factories [25]. It is its ability to retain dust during long-term work in industrial conditions that should be the subject of current research on filtration processes in the wood and wood processing industry.

The aim of the paper is to an experimental study of the separation efficiency of the filter fabric operating in two different industrial filters, considering the different service lives of filter bags made of this fabric. Moreover, an attempt to determine the influence of the bag working time in industrial conditions on its separation efficiency was made. Understanding this issue will allow the characterization of the nature of the filtration phenomenon in industrial conditions and the impact on it by the properties of waste, which, despite the constantly improved technology and conducted research [22,23,26,27,28,29,30,31,32,33] on dust extraction, still pass through the filter and pose the most significant air pollution hazard in the wood industry. Thus far, no studies have been carried out to determine the filtration efficiency of filter bags operating for a long time in industrial conditions. The obtained information will become the basis for determining the requirements for the use of non-woven filter fabrics for wood dust separation.

## 2. Materials and Methods

### 2.1. General Assumptions

The IKEA Industry furniture factory in Lubawa (Poland) was selected as the research site. The main reason for this choice is the very large scale of highly automated production, which for each type of processing has different technological solutions and dust extraction installations, which are an excellent source of obtaining a lot of information on the phenomena occurring during the separation of various types of dust waste from various wood composites machining operations. The tests were carried out for two different operating lines: surface treatment line and drilling centers line. Both lines are equipped with a JKF filter (Berzyna, Poland), in which the Gutsche filter bags (Fulda, Germany) were installed. Despite the fact that the same bags are assembled, both filters differ in design.

### 2.2. Samples Used for Testing

According to the assumptions, filter bags used in the IKEA Industry furniture factory in Lubawa were collected for the tests. Several new bags were inserted into the selected filters of two technological lines, which were removed at two-month intervals to perform the necessary experimental procedures. The bags, after being shortened to a length of 1500 mm, were installed on pilot-scale testing stand in the laboratory, and the separation properties of materials previously operated for a certain period in industrial conditions were tested. The list of the obtained samples of bags is presented in Table 1.

### 2.3. Narrow Surfaces Treatment Line of Furniture Panels

Two narrow surface processing sublines are connected to the dedusting installation. The main subline is a set of two machine tools that format and edge band furniture elements on both sides simultaneously. The machine tools have their own transport of elements, which are cut, formatted, and edge band at individual stages depending on the needs. Both machines on the line are separated by a turntable, thanks to which, after turning the elements by an angle of 90°, it is possible to process four sides of an element. The processing is carried out with circular saws and cutters. The line is adapted to producing elements made of particleboard, MDF, or solid wood. The auxiliary line processes one narrow surface of the bed joint and other narrow pieces of furniture. The same tools are used here as in the mainline. The total amount of waste generated on the line is 250 kg·h-1 with an average particle size of 140.88 µm. It is equipped with a two-part dust exhaust installation with one filter at the end. The production scheme on this line is described in the publication by Dembiński et al. 2021 [32].

### 2.4. Drilling Centers Line

At this point, a set of five numerically controlled drilling machines, used mainly for making structural holes in furniture elements, is connected to the dust extraction installation. Movable tilting spindles are also used for cutting curved elements with end mills. The machine tools are also equipped with a module for edge banding narrow surfaces of furniture elements. Dust with an average particle size of 168.64 µm in the amount of about 100 kg·h^−1^ is generated on the line of CNC drilling machines. The operation of the line was described in the publication by Dembinski et al. 2021 [34].

### 2.5. Characteristics of Dust Extraction Installations, Filters, and Filter Fabrics

Both the line for the narrow surface treatment and the line of drilling centers line are served by two installations connected to filters serving individual lines. Dust extraction installations for both narrow surface treatment lines and drilling center lines are equipped with JK-90 MT fans (Berzyna, Poland). Their chambers contain a different number of filter bags (140 pcs. In the filter in the extraction system on the narrow surface treatment line and 162 pcs. The air demand in dust extraction systems for each line is over 55,000. m^3^·h^−1^. The filtration speed value in the filter for the narrow surface treatment line is 4.918 cm·s^−1^ and 4.653 cm·s^−1^ in the filter for drilling center line. The filter bags were regenerated with an interval of 606 s in the filter for the narrow surfaces treatment line, while in the filter for the line of drilling centers line, this interval was 690 s. The dust load was respectively 4.509 g·m^−3^ for the narrow surface treatment line and 1.671 g·m^−3^ for the drilling centers line. The exact operating parameters of filters and dust extraction installations are described by Dembiński et al. 2021 [32]. Gutsche filter bags were installed in the described filters. The basic technical parameters of the material of filter bags are presented in Table 2.

### 2.6. Laboratory Research Stand

The filter bags obtained at the factory were tested on the stand for testing the filtration process on the pilot scale. The stand is used to determine the basic filtration parameters under set conditions. During the tests, the filtration parameters were maintained, corresponding to the actual conditions found in the industrial filter. In achieving these conditions, measurement and control systems were used:

- Dust dosing system in the intended amounts and concentration;
- Control valve for adjusting the volumetric capacity of the main air circulation fan;
- System controlling the frequency of regeneration of the filter element.

During the filtration at the testing stand, measurements of the number of dust particles at the outflow pipe were made at 5 min intervals. This measurement was performed using a Hiac/Royco 5250 A model laser particle counter (Rockford, IL, USA). Sampling was performed for 30 s five times during each filtration cycle. This device can automatically measure the number of particles in the purified air at the rated airflow through the measuring system equal to 2.831685 × 10^−2^ m^3^·min^−1^. Using a counter, you can determine the number of particles broken down into individual fractions from 0.5 to 25 µm. The counter shows the number of particles with the following sizes: 0.5; 1.0; 2.0; 3.0; 5.0; 10.0; 15.0; 25.0 µm. This gives the dimensional structure of the dust content in the purified air and further allows to determine the separation efficiency of the filter materials.

**Figure 1:**
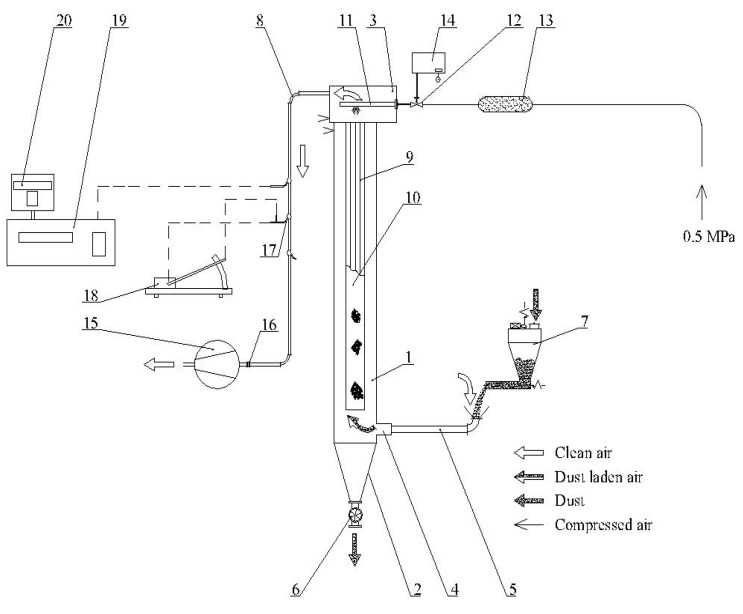
Test rig set-up: 1. filtering chamber, 2. hopper, 3. clean air chamber, 4. inlet, 5. dust inlet tube, 6. mucus feeder, 7. screw dust feeder DSK-I-04p (HYDRAPRESS, Białe Błota, Poland) 8. outflow pipe, 9. metal cage, 10. filtering bag, 11. cleaning nozzle, 12. electromagnetic valve, 13. compressed air tank, 14. The controlling device, 15. main fan, 16. gate valve, 17. Prandtl tube, 18. inclined-tube manometer type MPR-1 (ZAM Kety, Poland), 19. differential manometer type CMR-10 A (ZAM Kety, Poland), 20. printer.

The diagram of the laboratory stand operation is presented in Figure 1.

All bags were tested under the same controlled filtration conditions as presented in Table 3. The filtration parameters were selected based on previous tests carried out for wood dust corresponding to the operating conditions of industrial filtration [19,27,28,33,34,35,36,37].

The observed results were then compared with each other with regard to the working time of the bag.

### 2.7. Dust Used for Laboratory Tests

The bags were tested using test beech dust with a bulk density of 177.8 kg·m^−3^ from a bent furniture factory. The particle-size distribution of dust was determined by the sieve method using a Retsch AS200 (Haan, Germany) sieve machine.

### 2.8. Separation Efficiency

In order to determine the separation efficiency, the mass of dust particles in the air on both sides of the filter bag was determined. The mass of dust particles in the inlet dust in fractions corresponding to the size ranges for the dust content in the purified air was determined based on the setting of the dust feeder to the filtration chamber (10 g·m^−3^) and the determination of the particle size distribution of dust in sieve fraction < 0.032 mm carried out with the laser particle sizer Analysette 22 Micro-tec plus (Firtsch, Idar-Oberstein, Germany). The quantitative-dimensional structure of the dust in the incoming air to the bag in the test stand was determined concerning the assumed dimensional channels in accordance with the empirical particle distribution function, using the method described earlier in the study of Rogoziński (2016) [28] and presented in Table 4.

The separation efficiency of the tested filter bags was calculated for particles in the range of 0–25 μm. The dust content and the shares of its individual fractions in the purified air were determined based on the results of measurements carried out using the Hiac laser particle counter. The results obtained from the measurement of the number of particles per 1 m^3^ in the purified air were then converted into the mass of particles in individual fractions. The mass of dust particles in the filtered air in a given fraction per 1 m3 of air was determined according to Formula (1).

m_1i_ = V_cz_·m_sd_·a [kg] (1)

where:

V_cz_—dust particle volume [m^3^]

m_sd_—mass of wood substance (1500 kg·m^3^)

a—the number of particles in each size range obtained from a particle number measurement performed.

The volume of the dust particle *V_cz_* was calculated assuming that it takes the shape of a sphere with a diameter *D* equal to the arithmetic mean of the given size range in the measuring range of the particle counter according to Formula (2).

(2) $$V_{cz}=\frac{\pi }{6}D^{3}$$

After calculating the mass of particles in the purified air for individual fractions, their values were summed up to obtain the mass of dust of all 8 ranges in one cubic meter of purified air.

(3) $$m_{i}=\overset{8}{\underset{i=1}{\sum }}m_{1i}$$

The next step in the calculations was determining the separation efficiency for individual fractions in the filtered air. It was determined from the Equation (4).

(4) $$\eta_{i}=\frac{m_{0i}-m_{1i}}{m_{0i}}\cdot 100\%$$

where:

*η_i_*—separation efficiency for individual fractions

*m*_0*i*_—the mass of particles at the inlet to the filtration chamber of the given fraction

*m*_1*i*_—the mass of particles leaving the chamber for a given fraction

The final stage was to determine the total efficiency. It was determined according to Formula (5).

(5) $$\eta =\frac{\sum_{i=1}^{8}(m_{0i}-m_{1i})}{\sum_{i=1}^{8}m_{0i}}$$

## 3. Results and Discussion

**Figure 2:**
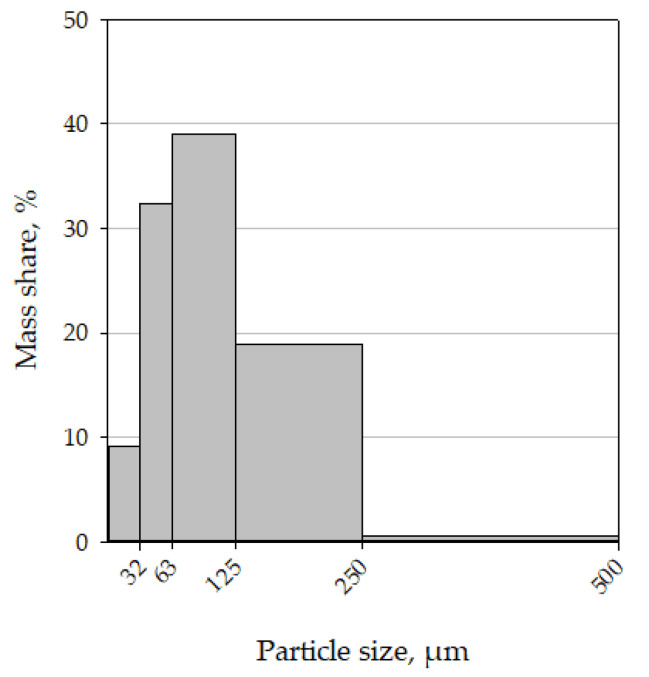
Dust particle-size distribution.

The particle-size distribution test was performed three times, and the result was aver-aged. The set of sieves used allowed for the separation of particles in size ranges from 0.00 to 0.032; from 0.032 to 0.063; from 0.063 to 0.125; from 0.125 to 0.500; and from 0.500 to 1.000 mm. The results are shown in Figure 2.

**Figure 3:**
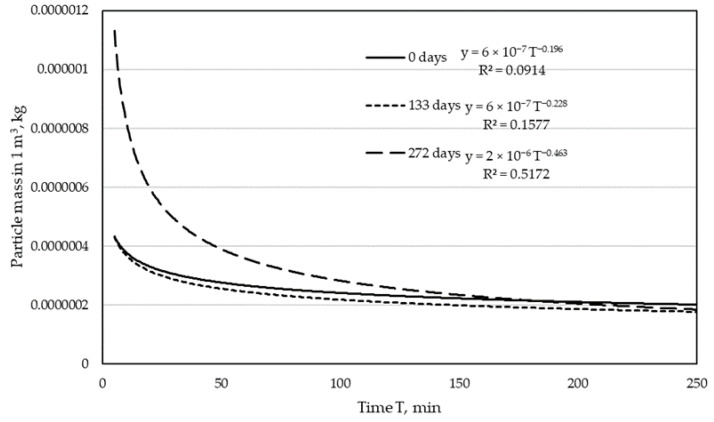
Particle mass in purified air for a drilling centers line.

**Figure 4:**
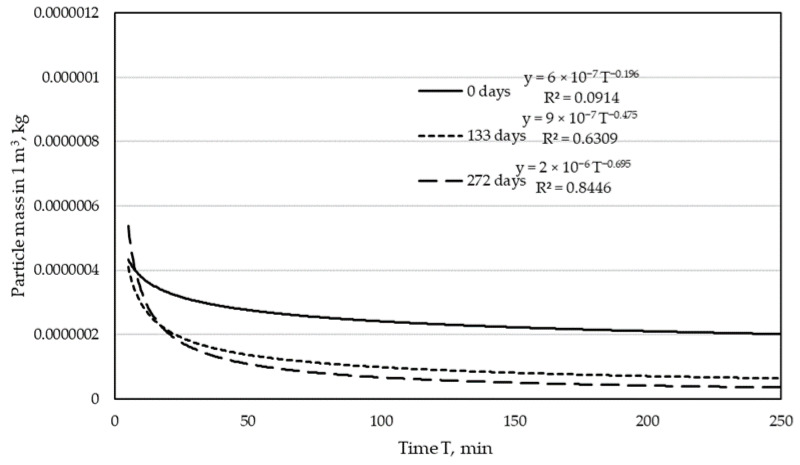
Particle mass in purified air for narrow surfaces treatment line.

Based on the Equation (3), the mass of particles in 1 m^3^ of purified air was determined. The calculation of this value is necessary to determine the separation efficiency of tested materials. The results for both lines are presented in the graphs (Figure 3 and Figure 4).

When analyzing the graphs in Figure 3 and Figure 4, it can be concluded that in the case of bags obtained from the filter in the installation connected to the drilling centers line (Figure 3), the total mass of particles in the purified air decreases with the extended period of industrial bag operation. The process is clearly different between a new bag and a bag that has been used for 133 and 272 days.

The analysis of the mass of particles in the purified air with the use of bags obtained from the filter in the dust extraction installation of the narrow surfaces treatment line (Figure 4) shows that the filtration process for a period of 133 days ran without significant changes in relation to the initial state in regards to the number of particles in the filtered air. The situation changed with the extension of the service life, and for 272 days, the mass of particles in the purified air decreased significantly in relation to the new bag. The decrease in the number of dust particles along with the bag operation time was found in their research by Dolny and Rogoziński in 2012 [27] and by Rogoziński [28]. The tests carried out by these authors confirmed that filtration time is one of the factors influencing filtration efficiency. These studies also showed a decrease in the filtration efficiency for particles that most penetrate the human respiratory tract, i.e., in the size (size) 2 and 3 µm.

The dependences presented in Figure 3 and Figure 4 show the influence of the operation time of the bag in the industrial dust collector on the mass of the test dust in the outlet air from the test stand. In the case of the bags from the filter of the line of drilling centers line, it is clearly visible that with increasing the operation time of the bags in the industrial dust collector, the mass of test dust in the purified air during the test systematically decreases. The decrease in dust mass is faster in the initial filtering period and gradually slows down. In Figure 3, we see a large difference in dust mass between a new and a used bag for 133 days. This difference is much smaller if the total mass of dust is compared between the used bags of 133 and 272 days. Therefore, it can be concluded that the dust mass retained in the filter in the first phase of filtration is greater than in the later periods of operation. The dust mass in the filtered air looks slightly different for the bags from the filter in the narrow surfaces treatment line (Figure 4). While in the first phase of filtration (133 days), a decrease in the dust mass in the outlet air is noticeable, the further use of the bag (up to 272 days) does not change this mass significantly.

**Figure 5:**
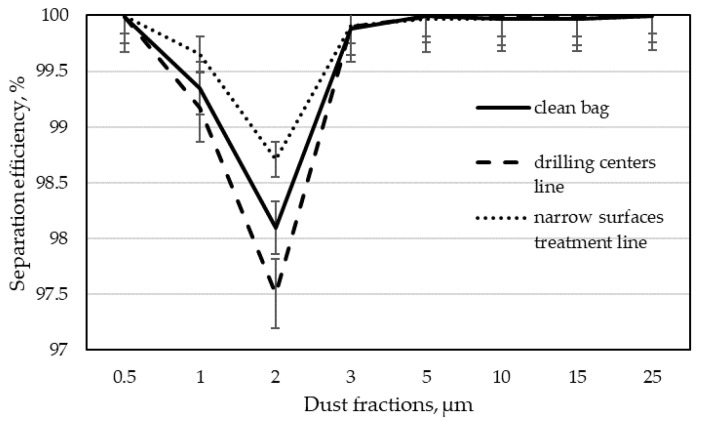
Fractional separation efficiency for 133 days of operation time of filter bags in individual dust extraction installations.

**Figure 6:**
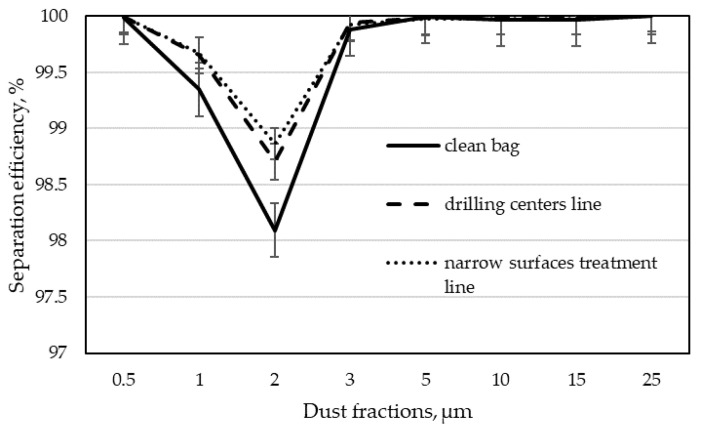
Fractional separation efficiency for 272 days of operation time of filter bags in individual dust extraction installations.

**Figure 7:**
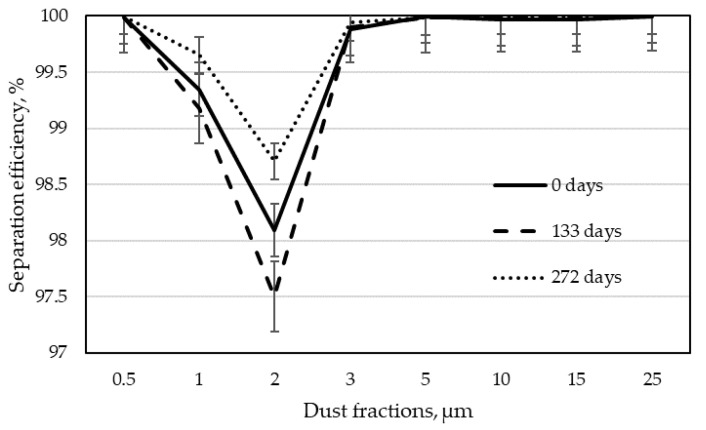
Fractional separation efficiency for filter bags from the dust extraction installations of drilling centers line in particular periods of use.

**Figure 8:**
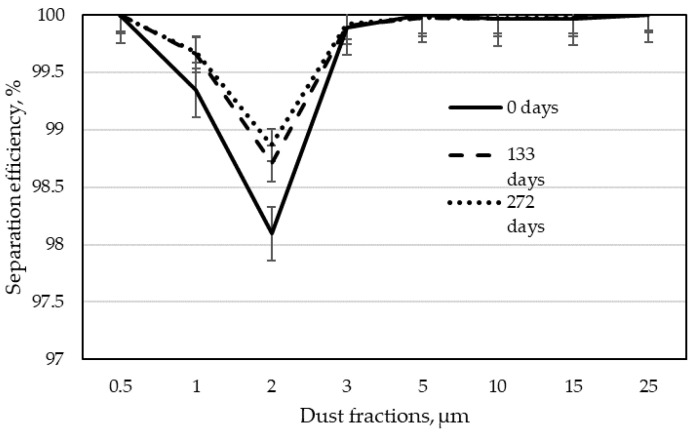
Fractional separation efficiency for filter bags from the dust extraction installation of a narrow surfaces treatment line in particular periods of use.

The next step in analyzing the results was to determine the separation efficiency for individual dust fractions according to Formula (3). By calculating the separation efficiency for each of the tested dust fractions, the dependence of this efficiency on the particle size was obtained. These dependencies are shown in Figure 5, Figure 6, Figure 7 and Figure 8 as graphs of fractional separation efficiency.

Laboratory tests have shown that the tested filter bags have the lowest separation efficiency for particles of 2 µm. It is a characteristic effect of shaping the separation efficiency of filter wood dust collectors using textile filter materials. A similar observation was found during their research by Rogoziński and Trofimov [33] and Rogoziński [35]. However, they showed that one of the critical factors influencing the efficiency of wood dust separation is air humidity. It has been shown that with increasing air humidity, the filtration efficiency rises. It has also been confirmed that thermal modification of the filter fleece surface increases filtration efficiency. Nevertheless, non-woven filter materials remain the least effective for particles of this size. 

A decrease in the separation efficiency for particles in the range of 2 to 5 µm was also demonstrated in the studies by Jackiewicz and Gradoń [22]. These researchers focused on increasing dust removal efficiency by using various non-wovens, showing that the use of thinner fibers or the use of the electrostatic effect in separation significantly increases its efficiency for aerosol particles. Xiao et al. [38] came to similar conclusions. They found that in addition to the thickness of the fiber, the filtration efficiency is also influenced by the increase in the surface density of the non-woven layer while maintaining the same fiber diameter and the pore size in the non-woven fabric. These studies, however, did not concern the influence of the aging of non-wovens on filtration efficiency.

The characteristic “V” shape on the separation efficiency chart for the 2 µm fraction was also obtained during the tests of two unused different filter fabrics (non-woven polyester fabric with an anti-clogging thermo-bonded surface and acrylic polymer microporous coating over a polyester non-woven fabric) [23]. This corresponded to the lowest separation efficiency for the most penetrating particles, i.e., depending on the non-woven fabric used, from 2 to 4 μm. Unfortunately, also, in this case, we are not dealing with testing non-wovens used in a long-term manner.

A decrease in the separation efficiency for particles with dimensions of 0.1–0.2 µm was also observed by other researchers. Balgis et al. [32] showed a similar relationship for the filtration of cellulose triacetate using a non-woven fabric with porous structures.

The study of separation efficiency in plywood production was presented by Welling et al. [5]. They considered wide-belt sanders connected to a common suction system with a 100% polyester filter fabric with a weight of 420 g·m^−2^. The study compared the separation efficiency of an industrial filter with various filtering materials during work in laboratory conditions with MDF dust. In addition to factory filter fabric (100% polyester filter with a weight of 420 g·m^−2^), glass fiber filters, glass microfiber, and paper filters were also tested. In this case, the lowest separation efficiency was recorded for dust in the range of 2 to 4 µm for the filter used in the factory. The remaining materials were approximately 90% effective for this particle size. The separation efficiency increased with increasing particle size. Unfortunately, these authors do not provide the duration of the bags’ operation in the factory, so their results cannot be directly compared to those presented in this work.

Graphs 7 and 8 show separation efficiency depending on the operation time of the non-woven fabric for individual wood dust fractions. It can be clearly seen that in the case of filter bags obtained from the narrow surfaces treatment line, the separation efficiency increases for all dust fractions with the increasing working time. The most significant increase in filtration efficiency over time was observed for the 2 µm particle size after 133 days. The separation efficiency of bags from the filter of the drilling centers line was slightly different. Here, in contrast to the filtration of dust from the narrow surfaces treatment line, the filtration efficiency for the most penetrating dust (2 µm) had the lowest values after 133 days. After 272 days of filtration, it increased, confirming the beneficial effect of filtration time on its efficiency. Such a result may indicate that the bag was mechanically damaged during operation in industrial conditions. It took a long time for the incoming dust to fill the damage and the separation efficiency for the most penetrating particles began to increase.

**Figure 9:**
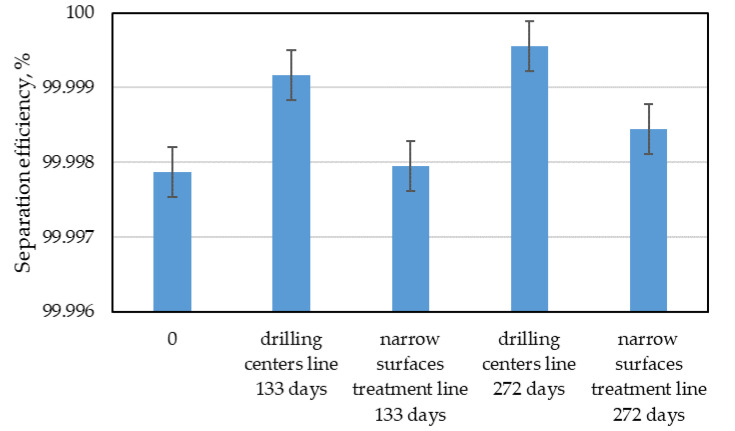
Total separation efficiency for all installations.

The final step was to determine the total filtration efficiency of the tested materials for both lines. The results were calculated on the basis of Formula (5) and presented in Figure 9. The results obtained for different working times of the filter materials were compared. In any case, the overall separation efficiency is very high. Similar results to those obtained in the research were obtained by Thorpe and Brown [24]. They examined the separation efficiency of beech dust generated when sanding wooden elements in simulated industrial conditions encountered in wooden furniture factories. These researchers showed that for hand sanders with external filter bags, the efficiency, depending on the granulation of the sanding paper used, ranged from 97.19 to 99.99%. Concerning the overall efficiency results, an analysis of variance (ANOVA) was performed. Thanks to it, it can be concluded that the use of the bag in the installation of the narrow surfaces treatment line for a period of 133 days does not show significant differences in separation efficiency compared to the control sample, i.e., a new, unused bag. Extending the operation time of the bags to 272 days in the same installation increased the separation efficiency, and similarity was found between its separation efficiency and the efficiency of other filters (narrow surfaces treatment line 133, drilling centers line 133, and drilling centers line 272). The bags from the dust extraction installations of CNC drills show a significantly higher filtration efficiency than the other tested ones.

## 4. Conclusions

The conducted tests have shown that the filtration efficiency of bags operating in industrial conditions depends on the filtration conditions as well as the amount and properties of dust. The dust mass in the purified air decreases with the duration of use of the filter bags. 

The analysis of the fractional dust separation efficiency clearly showed that for very small (0.5 and 1 µm) and large particles (over 3 µm), the efficiency reaches very high values. The least effective was for particles with a size of 2 µm.

It was also found that for the bags from both dust extraction installations, separation efficiency is higher with longer use of the bag, and so for the extraction installation in the line of CNC drilling machines, the efficiency increased earlier and to a higher level than for the filter of the installation in the narrow surfaces treatment line.

## Figures and Tables

**Table 1:** Test bags obtained from the furniture factory.

Dust Exhaust Installation	Bag Working Time [Days]	Bag Producer
Narrow surfaces treatment line	0, 67, 133, 272	Gutshe
Drilling centers line		

**Table 2:** Basic technical parameters of the filter bags used according to the manufacturer.

Parameter	Unit	Parameter Value
Bag producer		Gutshe
Material type/symbol		Polyester with PP film
Material weight	g.m^−1^	400
Material thickness	mm	1.5
Tensile strength—lengthwise	daN·5 cm^−1^	40
Tensile strength—across	daN·5 cm^−1^	50
Air permeability	dm^3^·min^−1^·dm^−2^	250
Surface finishing		Thermal stabilization, calendering
High-temperature resistance	°C	90
Acid resistance		Good
Alkali resistance		Sufficient
Water-resistant		Weak
Declared filtration efficiency for particles > 2.5 µm	%	99.998
Declared filtration efficiency for particles < 2.5 µm	%	99.957

**Table 3:** Filtration parameters during testing of filtering non-wovens.

Parameter	Unit	Parameter Value
Maximum airflow velocity in the main fan duct (Figure 1 p. 15) *w*	m·s^−1^	4.290
Average velocity $\bar{w}$ = 0.85 *w*	m·s^−1^	3.646
Air volume flow V	m^3^·s^−1^	0.0286
	m^3^·h^−1^	103.0
Air to cloth ratio *f*	m^3^·(m^2^·h)^−1^	145.8
Filtration velocity *w_f_*	m·s^−1^	0.0405
Dust concentration	G·m^−3^	10

**Table 4:** Dust mass in the in 1 m^3^ of inlet air.

Dimensional Range [µm]	Percentage [%]	Inlet Fraction Mass [g]	Inlet Fraction Mass [kg]
<0.5	0.173983	0.0173983	1.73983 × 10^−5^
0.5–1	0.035953	0.003595325	3.59533 × 10^−6^
1–2	0.008763	0.00087631	8.7631 × 10^−7^
2–3	0.007876	0.000787628	7.87628 × 10^−7^
3–5	0.100016	0.010001631	1.00016 × 10^−5^
5–10	0.703310	0.070331044	7.0331 × 10^−5^
10–15	0.980705	0.098070539	9.80705 × 10^−5^
15–25	1.906999	0.190699982	0.0001907
Total Dimensional range from 0.5 µm to 25 µm		0.391760759	0.000391761
more than 25 µm	96.082392	9.608239241	0.009608239

## Data Availability

Not applicable.
